# Profiling of serum and tissue high abundance acute-phase proteins of patients with epithelial and germ line ovarian carcinoma

**DOI:** 10.1186/1477-5956-6-20

**Published:** 2008-07-18

**Authors:** Yeng Chen, Boon-Kiong Lim, Suat-Cheng Peh, Puteri Shafinaz Abdul-Rahman, Onn Haji Hashim

**Affiliations:** 1 Department of Molecular Medicine, Faculty of Medicine, University of Malaya, 50603, Kuala Lumpur, Malaysia; 2 Department of Obstetrics & Gynecology, Faculty of Medicine, University of Malaya, 50603, Kuala Lumpur, Malaysia; 3 Department of Pathology, Faculty of Medicine, University of Malaya, 50603, Kuala Lumpur, Malaysia

## Abstract

**Background:**

Acute-phase response involves the simultaneous altered expression of serum proteins in association to inflammation, infection, injury or malignancy. Studies of the acute-phase response usually involve determination of the levels of individual acute-phase serum proteins. In the present study, the acute-phase response of patients with epithelial (EOCa) and germ-line (GOCa) ovarian carcinoma was investigated using the gel-based proteomic approach, a technique which allowed the simultaneous assessment of the levels of the acute-phase serum high abundance proteins. Data obtained were validated using ELISA and immunostaining of biopsy samples.

**Results:**

Enhanced expression of clusterin (CLU), α_1_-antitrypsin, haptoglobin and leucine rich glycoprotein was detected in all patients. However, the levels of α_1_-antichymotrypsin (ACT) was only enhanced in EOCa patients, while patients with GOCa were typically characterized by elevated levels of ceruloplasmin but lower levels of α_2_-HS glycoprotein. The enhanced expression of CLU in EOCa and GOCa patients and up-regulated expression of ACT specifically in EOCa patients were confirmed by ELISA. Immunohistochemical staining of biopsy samples of EOCa and GOCa patients demonstrated correlation of the acute-phase protein expression.

**Conclusion:**

Patients with EOCa and GOCa demonstrated distinctive aberrant expression of serum and tissue high abundance acute-phase proteins compared to negative control women.

## Background

The expression of serum proteins can be analysed concurrently by using the gel-based proteomic technology. This is appropriate for studying the acute-phase response, which involves the simultaneous altered expression of serum proteins in association to inflammation, infection, injury or cancer [1]. Many of the proteomic studies on serum or plasma have been performed using samples that were depleted of albumin and/or immunoglobulins in order to analyze serum proteins of lower abundance [2-4]. However, a number of serum proteins including those that have been used clinically or experimentally have been demonstrated to adhere strongly to albumin and immunoglobulins [5]. These serum proteins were also removed in experiments involving depletion of the high abundance proteins, and thus, may affect interpretation of the results. Moreover, recent studies using rat plasma have revealed that depletion of high abundance proteins only reduced the dynamic range of plasma proteome by two to three orders of magnitude. Removal of albumin, IgG, IgM, transferrin, fibrinogen, haptoglobin (HAP) and α_1_-antitrypsin (AAT) from rat plasma leads to the unmasking of only a few proteins and was still far from being able to detect the low abundance proteins [6].

Our previous gel-based proteomic studies performed on unfractionated whole serum samples of patients with different types of cancer have highlighted the altered expression of selective high abundance acute-phase reactant proteins. Breast cancer patients were reflective of their differential expression of serum α_1_-antichymotrypsin (ACT), clusterin (CLU) and complement factor B (CFB) [7], while patients with nasopharyngeal carcinoma expressed the sole elevated levels of serum ceruloplasmin (CPL) [8]. Although the expression of AAT, α_1_-B glycoprotein (ABG) and anti-thrombin III were consistently altered in patients with endometrial adenocarcinoma (EACa), squamous cell cervical carcinoma (SCCa) and adenocervical carcinoma (ACCa), CLU was specifically up-regulated in patients with EACa, whereas patients with SCCa and ACCa were typically characterized by the up-regulated expression of zinc α-2-glycoprotein (ZAG) [9].

In the present study, we have analyzed the expression of high abundance acute-phase reactant proteins in sera of patients who were newly diagnosed with epithelial ovarian carcinoma (EOCa) and germ line ovarian carcinoma (GOCa) using the gel-based proteomic approach. The expression of the proteins was validated using ELISA as well as by immunohistochemical staining of cancer tissues from the patients.

## Results

### Serum Protein Profiles

**Figure 1:**
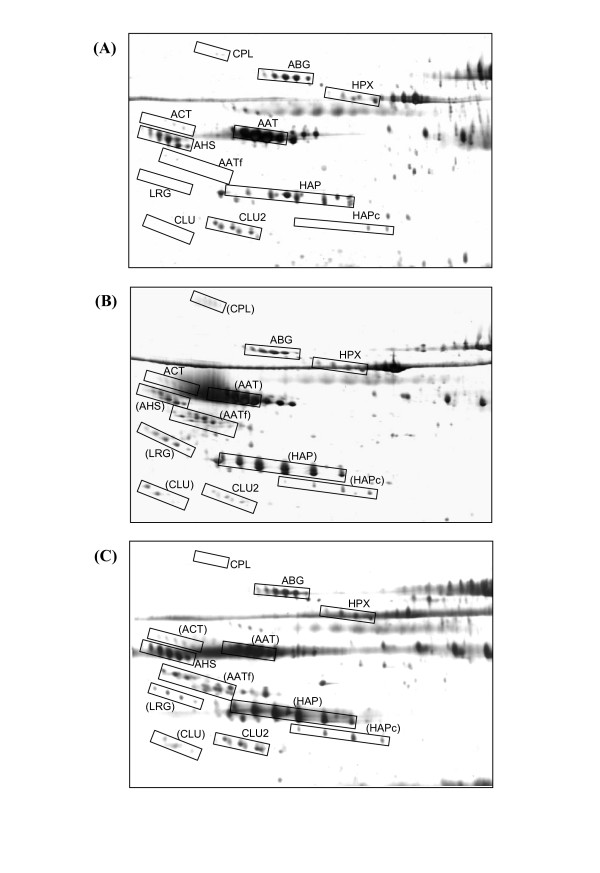
**Typical 2-DE serum protein profiles of the negative control women and patients with GOCa and EOCa**. Unfractionated serum samples of patients and negative controls were subjected to 2-DE and silver staining. Panel A demonstrates a typical representative 2-DE serum protein profile of negative control women. Panels B and C demonstrate typical representative unfractionated serum protein profiles of the patients with GOCa and EOCa, respectively. The aberrantly expressed proteins are marked in brackets for panels B and C. For all panels, the acidic sides of the 2-DE gels are to the left and relative molecular mass declines from the top.

When unfractionated whole sera of negative control women unaffected by cancer (n = 30) were subjected to 2-DE and silver staining under the resolving conditions adopted in the present study, the high abundance proteins that were detected include albumin, the heavy and light chains of IgA, IgG and IgM, two groups of CLU (CLU and CLU2), AHS, ABG, AAT and its fragment AATf, ACT, CPL, β chains of HAP (HAP), leucine rich glycoprotein (LRG) and hemopexin (HPX) (Figure 1, panel A). When the 2-DE experiments were performed on sera of 42 patients with ovarian carcinoma (n = 13 for GOCa and n = 29 for EOCa) who were newly diagnosed and untreated, comparable profiles were obtained for most of the resolved proteins. Panels B and C of Figure 1 demonstrate typical 2-DE serum protein profiles of patients with GOCa and EOCa, respectively. In both subtypes of the ovarian carcinoma patients, three additional clusters of proteins including CLU, cleaved β chains of HAP (HAPc) and a different cluster of AATf spots were detected.

### Identification of Serum Proteins

With exception of LRG, all the other serum high abundance clusters of protein spots have been previously identified by mass spectrometry and/or protein sequencing [7-10]. In cases of AAT and CLU, different forms of the serum proteins (AATf and CLU2) were also detected in the present study. Identities of LRG, AATf and CLU2 were confirmed by subjecting the protein spot clusters to MALDI-MS analysis and database search (Table 1). Some of the AATf spots within the cluster were identified using MALDI-MS/MS with 28 sequences of peptides correlating to the protein.

**Table 1:** MS identification of protein spot clusters from 2-DE serum protein profiles.

**Serum** ** proteins^*a*^**	**Accession** ** number^*b*^**	**Theoretical** ** M_r _(da)/p*I***	**No. of peaks** **matched (No. of** ** peaks searched)**	**MASCOT** ** score**	**% of ** **sequence** ** coverage**
LRG	gi | 112892	38178/6.45	8(11)	67	22
CLU2	gi | 4502905	4877/6.27	6(10)	50	16
AATf	gi | 1703025	46707/5.37	8(14)	68	20
AATf ^*c*^	gi | 7546268	37565/5.5	28^*d*^	307	50

*a *Spot ID are as in Figure 1. M_r_: nominal mass

*b *Accession numbers are from the MASCOT database

*c *Spots within cluster identified by MALDI-MS/MS analysis

*d *Number of peptides matched

### Image Analysis of 2-DE Gels

**Figure 2:**
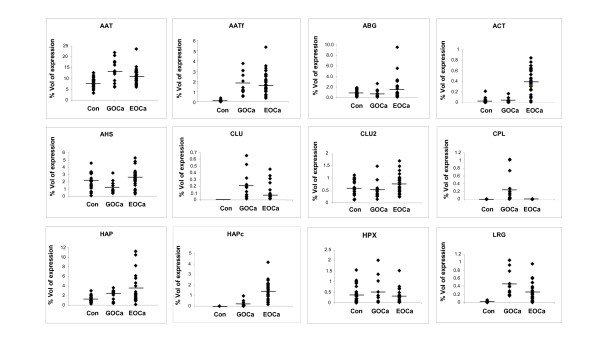
**Percentage of volume contribution of 2-DE detectable serum proteins of control women (Con) and patients with EOCa and GOCa**. Percentage of volume contribution (%vol) of protein spots was analyzed by ImageMaster™ 2D Platinum Software Version 5. Analysis was restricted to twelve clusters of protein spots.

When the clusters of protein spots were analyzed by a densitometry software, higher expression of CLU (*p *< 0.0001), AAT (*p *< 0.001), AATf (*p *< 0.0001), LRG (*p *< 0.0001), CPL (*p *< 0.05), HAP (*p *< 0.0005) and HAPc (*p *< 0.005) was detected in sera of patients with GOCa (Figure 2). However, the expression of AHS (*p *< 0.05) appeared to be lower in GOCa patients compared to the negative controls. There was no significant difference in the expression of CLU2, HPX and ABG between the GOCa patients and the negative controls.

Similar analysis performed on serum images of patients with EOCa also revealed the aberrant expression of serum proteins. Higher expression of ACT (*p *< 0.0001), CLU (*p *< 0.005), AAT (*p *< 0.0001), AATf (*p *< 0.0001), LRG (*p *< 0.0001), HAP (*p *< 0.0005) and HAPc (*p *< 0.0001) was significantly detected (Figure 2). There was no significant difference between the expression of AHS, CLU2, HPX and ABG of patients with EOCa compared to the negative controls.

### Analysis of Protein Expression by ELISA

**Figure 3:**
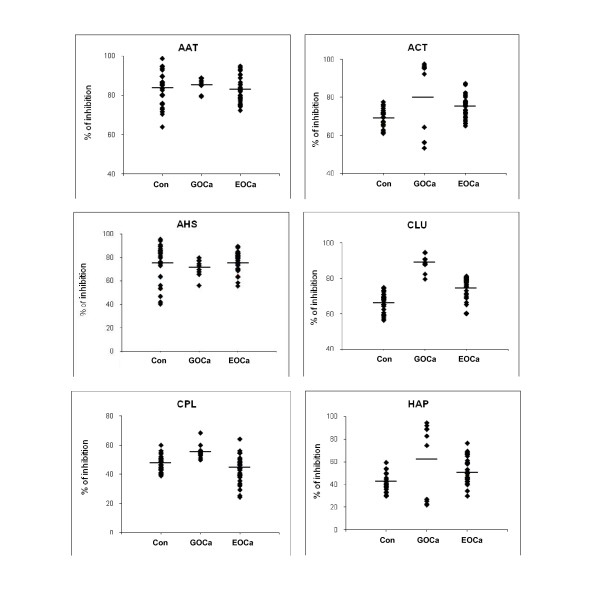
**Analysis of serum protein expression by competitive ELISA**. Competitive ELISA was preformed using sheep/goat anti-human ACT, AHS, CLU, CPL, AAT and HAP as primary antibodies. Analyses were performed in triplicate. Relative expression of serum proteins in GOCa and EOCa patients is defined as percentage (%) of up- or down regulation from that of negative controls.

For confirmation of the aberrantly-expressed proteins in the sera of GOCa and EOCa patients, competitive ELISA was carried out using antisera against ACT, CLU, CPL, AHS, AAT and HAP. Figure 3 demonstrates the results of the competitive ELISA performed on sera of negative control women, GOCa patients and EOCa patients. Higher levels of CLU (*p *< 0.0001) were significantly detected in sera of the GOCa patients compared to the negative control women. In the case of EOCa patients, the expression of CLU (*p *< 0.0001), ACT (*p *< 0.0001) and HAP (*p *< 0.05) was significantly higher than that of the negative controls. However, the expression of AAT, AHS and CPL in both cohorts of patients was not significantly different from that of the control women.

### Analysis of Tissue Protein Expression by Immunohistochemical Staining

**Figure 4:**
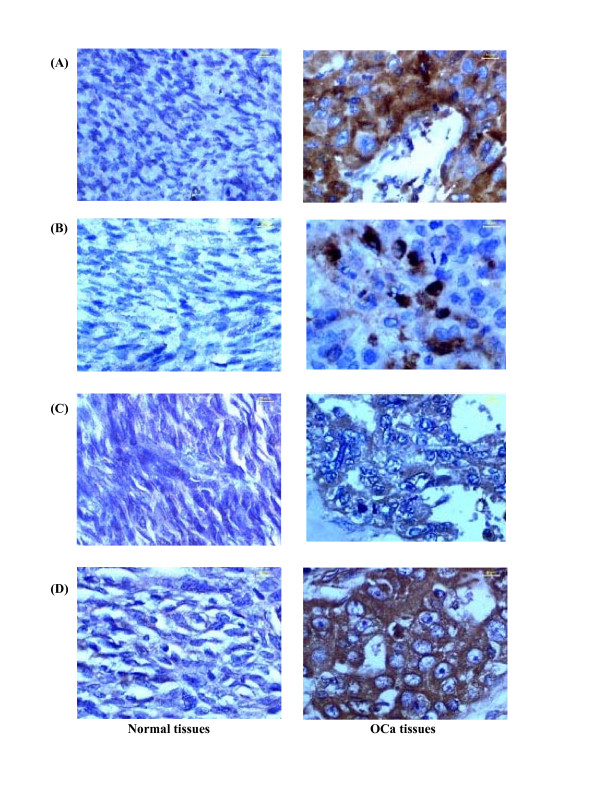
**Immunohistochemical analysis of protein expression in ovarian tissues of normal donors and ovarian carcinoma patients**. Immunohistochemical staining was performed using antisera to (A) CPL, (B) CLU, (C) ACT, and (D) AAT. Panels A and B demonstrate immunohistochemical stains showing strong positive CPL and CLU expression in ovarian cancer (OCa) tissues but negative expression in normal ovarian tissues (internal positive control). Panels C and D demonstrate immunohistochemical stains showing moderate positive ACT and strong positive AAT expression in OCa tissues, respectively, but weak positive expression in normal ovarian tissues.

The expression of CPL, CLU, ACT and AAT was determined for 17 biopsy samples of patients with ovarian carcinoma. Among 17 tissue samples, two were from patients diagnosed with GOCa and 15 were from patients diagnosed with EOCa. The overall results and staining patterns of the biopsy samples of patients with clinically- and histopathologically-confirmed ovarian carcinoma are summarized in Table 2. The expression of CPL and CLU was positively-stained consistently in all ovarian carcinoma tissues that were studied. However, ACT was positively-stained in 13 biopsy samples (two cases of GOCa and 11 cases of EOCa), while positive-staining for AAT was detected in ten biopsy samples (one case with GOCa and nine cases with EOCa) (Figure 4).

**Table 2:** Analysis of protein expression by immunohistochemical staining of patients' biopsy samples.

**Antibody**	**GOCa**	**EOCa**	**Total**
CPL	2/2	15/15	17/17
CLU	2/2	15/15	17/17
ACT	2/2	11/15	13/17
AAT	1/2	9/15	10/17

## Discussion

EOCa and GOCa are two different types of ovarian cancer that affect women of different age groups. While EOCa is more common in pre-menopausal women, GOCa is predominantly a cancer that affects adolescents and young women under 30 years of age. EOCa arises from the epithelium or outer cells covering the ovary and it accounts for more than 90% of ovarian cancer cases. GOCa differs from EOCa in that it arises from the cells that mature into eggs that are formed inside the ovary [11].

By using 2-DE and image analysis, we have demonstrated the alteration of serum high abundance proteins expression involving higher expression of CLU, AAT, AATf, LRG, HAP and HAPc in sera of both cohorts of patients with EOCa and GOCa. However, the expression of ACT was only enhanced in EOCa patients while patients with GOCa demonstrated lower levels of AHS and enhanced expression of CPL compared to the negative controls. Since the controls used in the present study were serum samples obtained from normal healthy individuals, we cannot totally exclude the possibility that the aberrant protein expression may be due to inflammation of the ovary rather than cancer. Nevertheless, similar preliminary experiments performed on patients with inflammation of the ovary indicated that the high abundance acute-phase protein expression was markedly different from those of the EOCa and GOCa patients (data not shown).

The elevated levels of CLU in both cohorts of EOCa and GOCa patients and enhanced expression of ACT only in patients with EOCa were confirmed by ELISA. ELISA also confirmed the up-regulated expression of HAP in EOCa patients but was not able to demonstrate the difference in the expression of HAP and CPL in patients with GOCa as well as the up-regulated expression of AAT in both cohorts of the ovarian carcinoma patients. CLU and CPL were positively-detected in the biopsy samples of all patients tested while staining of ACT was positive in 13/17 patients' tissues analyzed.

CLU is implicated in diverse physiological functions including the regulation of cell growth and survival [12]. Its enhanced expression has been described in breast cancer [8], hepatocellular carcinoma [13], desmoplastic melanoma [14], colorectal cancer [15], bladder cancer [16], lymphomas [17] as well as ovarian cancer [18]. Multiple isoforms of CLU, arise from various post-translational modification processes, have been detected [19]. In the present study, two groups of CLU were detected in the 2-DE protein profiles. While the main group of CLU was up-regulated in the sera of EOCa and GOCa patients, CLU2 was not abnormally expressed. These results are compatible with the recent findings of Rodriguez-Pineiro and coworkers in their studies on colorectal cancer [15]. Our data also emphasized the expression of CLU in the malignant tissues of all ovarian carcinoma patients analyzed.

Like CLU, CPL is another protein reported to be elevated in sera of patients with diverse types of cancer. Elevated levels of CPL has been described in studies on thyroid cancer [20], melanoma [21], lymphoma [22], kidney and urinary tract cancer [23], gastrointestinal cancer [24], cervical cancer [9,25], uterine cancer [9,26] and nasopharyngeal carcinoma [8]. When bound to copper, CPL induces angiogenesis, which supports growth of tissues and tumors [27]. In the present study, detection of CPL in tissues of all patients tested is indicative of the strong association of angiogenesis with malignancy. The up-regulated expression of CPL was also significantly detected by 2-DE in the GOCa patients but not in patients with EOCa.

ACT, a member of the serine proteinase inhibitor family, is an acute-phase response protein [28]. Its association with cancer is rather restrictive unlike CLU and CPL. Previous studies have shown that the levels of ACT were also elevated in patients with breast cancer [7,29], and the excessive ACT was likely to be synthesized by the breast epithelial cells [30,31]. Hence, ACT may be also synthesized by the epithelial cells of the ovary which explains its observed up-regulated expression in sera of patients with EOCA but not GOCa patients both by using 2-DE and ELISA. In the tissues of the ovarian cancer patients, however, ACT was positively-stained in both the two biopsy samples of GOCa patients tested as well as 76% of the biopsy samples of EOCa patients.

The enhanced levels of AAT in sera of ovarian carcinoma patients that were detected in our 2-DE studies were not supported by the results of our ELISA experiments. Nevertheless, the up-regulated expression of AAT, which was always linked to liver diseases [32], as well as its N-terminal fragment, has been previously reported in the sera of patients with cancers of the prostate, lung and breast [33] although this association was not well understood. Similarly, our 2-DE experiments also detected the up-regulated expression of AATf, which were likely to be generated by the proteolysis of AAT, in both EOCa and GOCa patients. This appears to indicate that AAT was actively processed within the cancer microenvironment. AAT was also positively-detected in 59% biopsy samples of patients analyzed, inclusive of both subtypes of ovarian carcinoma.

Like AAT, ELISA was not able to confirm the different altered expression of AHS and HAP in sera of ovarian carcinoma patients by 2-DE studies. Our previous reports have indicated that the 2-DE proteomic approach is more sensitive than ELISA with many of the proteins analysed showed more than ten-fold difference in sensitivity between the two techniques [7,8]. One possibility for the difference in sensitivity as well as the discrepancy in the results is that the up-regulated proteins may have triggered the humoral immune response in patients and caused binding of the patients' antibodies to the serum proteins. Such binding would certainly interfere with the ELISA and reduce its sensitivity.

The correlation between HAP expression and ovarian carcinoma has been widely reported [34,35]. Studies have also shown both the α- and β-subunits of HAP to be significantly increased in early stage ovarian carcinoma patients [36,37]. In the case of AHS, our data showed down-regulation of the protein in sera of both cohorts of EOCa and GOCa patients. The low concentration of AHS in ovarian carcinoma serum may be associated with depressed cellular immunity [38] and short-term mortality of patients similar to that reported for patients with liver cancer [39].

Due to the lack of antiserum, we were not able to validate the results of the 2-DE experiments on overexpression of LRG in sera and tissues of EOCa and GOCa patients. LRG, a secretory protein of unknown function [40], has been noted for its acute-phase protein characteristics [41]. We have also recently reported the up-regulated expression of LRG in sera of patients with endometrial adenocarcinoma [9].

Our present data obtained from patients with ovarian carcinoma, when taken together with our previous reported studies on sera of patients with cancers of the breast [7], nasopharynx [8], cervix and endometrium [9], demonstrated that the cancer patients were aberrant in the expression of their serum high abundance acute-phase proteins, and this was simultaneously detected using the gel-based proteomic analysis on whole human serum samples without the need to deplete albumin or immunoglobulins. Equally intriguing is that the different types of cancer that were studied so far appear to demonstrate distinctive altered patterns of serum high abundance protein expression. In the case of ovarian carcinoma, this explicitly involved elevated levels of CLU in both the EOCa and GOCa patients and enhanced expression of ACT only in patients with EOCa.

The different altered expression of selective high abundance acute-phase serum proteins of patients with EOCa and GOCa suggests that they may be used as protein signatures that may facilitate diagnoses and/or monitoring of the cancers. Ovarian carcinoma is usually asymptomatic in its early stages and development. For most patients, the disease is often widespread at the time of diagnosis, and this is also due to the absence of sensitive and reliable serological markers. CA125, the currently accepted serum marker for diagnosis of ovarian carcinoma, is limited in sensitivity as it is detected in about 50% of patients at stage 1 and 75–90% of women with advanced stage of the disease. Moreover, it lacks specificity as it is also elevated in 26% of non-ovarian malignancies, 14% benign ovarian disease and 9% of benign gynecologic disorders [42]. Thus the need for more specific and efficient biomarkers for diagnosis of ovarian carcinoma is pressing. Although limited in scale, identification of the link between selective aberrantly expressed serum proteins with EOCa and GOCa as demonstrated in the present gel-based proteomic study is a necessary prelude to the conduct of a larger scale clinical investigation in pursuit of additional diagnostic markers that are more reliable and sensitive.

## Conclusion

The present study demonstrated that the serum and tissue high abundance acute-phase proteins were aberrantly expressed in patients with EOCa and GOCa. In contrast to control individuals, enhanced expression of CLU, AAT, HAP and LRG was detected in all patients. However, the levels of ACT was only enhanced in EOCa patients, while patients with GOCa were typically characterized by elevated levels of CPL but lower levels of AHS. These profiles were different from those previously obtained from patients with cancers of the breast, nasopharynx, cervix and endometrium.

## Methods

### Subjects

Blood specimens were obtained in plain tubes from 29 patients with EOCa (ages between 24 to 65 years) and 13 GOCa patients (ages between 12 to 24 years) at the University of Malaya Medical and Specialist Centres. From the 29 EOCa patients, four were diagnosed with stage 1, ten with stage 2, twelve with stage 3 and three with stage 4 of the cancer. Only one of the GOCa patients was diagnosed with stage 4 of the disease. Two patients were diagnosed with stage 1, four with stage 2 and six with stage 3 of GOCa. Samples were immediately centrifuged at 3000 *g *for 10–15 minutes at 4°C. The supernatant (serum) was collected and kept in aliquots of 50 μl. Control samples were obtained from 30 voluntary women without cancer. The expression of high abundance proteins in sera of control women of ages between 12 to 24 years (n = 10) was found to be comparable to that of the control women between the age of 24 to 65 years (n = 20) (data not shown). Samples obtained were with consent and approval granted by the Ethical committee (Institutional Review Board) of the University of Malaya Medical Centre in accordance to the ICH GCP guideline and the Declaration of Helsinki.

### Two-dimensional gel electrophoresis

2-DE was performed as previously described [7-9]. Briefly, 10 μl of unfractionated whole human serum samples were subjected to isoelectric focusing in 11 cm rehydrated precast immobilized dry strips pH 4–7 (GE Healthcare Bio-Sciences, Uppsala, Sweden). For the second dimension, focused samples in the strips were subjected to electrophoresis using the 8–18% gradient polyacrylamide gel in the presence of sodium dodecyl sulfate (SDS-PAGE). All samples were analyzed in duplicate.

### Coomassie Blue and silver staining

The 2-DE gels were developed by silver staining as described by Heukeshoven and Dernick [43]. For mass spectrometric analysis, gels were stained with Coomassie Blue according to the modified method of Shevchenko *et al*. [44]. Coomassie Blue was used in this case as higher number of peptides was recovered from the in-gel digestion of Coomassie stained gel plugs compared to the plugs from silver-stained gels.

### Mass spectrometry

Highly resolved spot clusters of proteins of interest were preliminarily identified by visual comparison with the SWISS ExPASy standard plasma protein reference [45] before being subjected to precise identification by mass spectrometry using the Ettan MALDI-ToF Pro. In-gel trypsin digestion was performed according to the method of Shevchenko *et al. *[44]. Mass analyses were performed by mixing peptide solutions with matrix solution consisting of 10 mg/ml α-cyano-4-hydroxycinnamic acid in 0.5% trifluoroacetic acid (TFA) and 50% acetonitrile (ACN) in a ratio of 1:1. The mixture (0.3 μl) was then applied onto the sample slide. Spectrum was calibrated using peptides included in the matrix solution, approximately 3 pmol of ^Ile7^Ang III and hACTH 18–39 with the expected m/z at 897.523 [(M + H)^+^, monoisotopic] and 2465.191 [(M + H)^+^, monoisotopic], respectively. Protein spots that were not positively identified were subsequently sent for MS/MS analysis using a MALDI-ToF/ToF instrument (Applied Biosystem 4700 Proteomic Analyzer) at the Australian Proteomic Analysis Facility (APAF), Macquarie University, Sydney, Australia. Gel plugs were kept in fresh eppendorf tubes containing small volumes of milliQ water to ensure that they remained hydrated prior to analysis.

### Database searches

Peaklist data obtained from PMF and MS/MS analyses were generated using the Ettan MALDI software (release version 2.0) and 4000 Series Explorer software (release version 3), respectively. The data were exported to the MASCOT search engine (Matrix Science Ltd., London, UK; release version 2.2). Search was performed against all entries in the NCBI non redundant database (updated April 25, 2007, containing 192489 sequences). The following parameters were used in the MASCOT PMF search: (i) enzyme: trypsin, (ii) one missed cleavage allowed, (iii) taxonomy limited to *Homo sapiens*, (iv) mass value: monoisotopic, (v) peptide mass tolerance: ± 0.1 Da and (iv) peptide charge state: 1+. The same parameters were used in the MASCOT Ion Search, except peptide mass tolerance and fragment mass tolerance were set at 50 ppm and 0.5 Da, respectively. The cut-off score for accepting individual MS/MS spectra was >29 to indicate homology and >38 for indication of identity.

### Image analysis

The LabScan image scanner (Version 5) was used to capture and store images of 2-DE gels. The ImageMaster™ 2D Platinum Software (Version 5) was used to evaluate the protein profiles and present information obtained from the 2-DE gels. To detect proteins that were differentially expressed in sera, percentage of volume contribution (%vol), which refers to the volume percentage of a protein taken against the total spot volume of all proteins including the unresolved peptides in each gel, was calculated. Data obtained in such expression are independent of variations attributed to protein loading and staining.

### ELISA

Competitive ELISA was performed as previously reported [7-9]. Concentrations of CPL, CLU, AAT, ACT, HAP and α_2_-HS glycoprotein in serum samples were reflected by their ability to inhibit the specific binding of antisera. Primary antisera (IgG fraction) comprised goat anti-human CLU (Chemicon International, Temecula, California, USA) and sheep anti-human ACT, AAT, AHS, CPL and HAP (The Binding Site, Birmingham, UK). Peroxidase conjugated donkey anti-sheep and goat IgGs (The Binding Site, Birmingham, UK) were used as the secondary antisera. Assay was not performed for LRG as antiserum to the protein was not available commercially. Enzyme activity was revealed by addition of 3,3',5,5' tetramethylbenzidine (Pierce, Rockford, Illinois, USA). Levels of proteins in the test sera are proportional to the percentage inhibition of substrate hydrolysis.

### Immunohistochemical staining

A total of 17 tissue specimens, retrieved from tissue archives (as paraffinized tissue blocks), were selected for histopathology analysis. Two were from patients with GOCa and 15 from confirmed EOCa specimens. Embedded tissue sections that were mounted on slides contained normal and cancerous tissues. Staining of tissues was performed as previously described [7,8]. Both primary and secondary antibodies were similar to that used in ELISA. The intensity of the positive staining was compared to that of the negative control tissues (substitution of the primary antibody with buffer) and divided into three levels of intensity of expression, with diminishing scale from 3+ to 2+ and 1+. Cases were considered highly positive (3+) when cells showed strong intensity, moderately positive (2+) when more than 10% of the cells were stained and weakly positive (+1) if less than 10% of the cells were stained. Negative (-) staining was indicated for those cells without any immunoreactivity.

### Statistical analysis

Levels of proteins in the gels are presented as mean %vol ± SD (standard deviation). The Normal test (Z) was used to analyze the significance of differences between control subjects and patients and to examine the correlation between the variable. *P*-values of less than 0.05 (p < 0.05) were considered statistically significant.

## Abbreviations

**AAT**: α_1_-antitrypsin; **AATf**: Fragment of AAT; **ABG**: α_1_-B glycoprotein; **ACT**: α_1_-Antichymotrypsin; **AHS**: α_2_-HS glycoprotein; **CLU/CLU2**: Clusterin; **CPL**: Ceruloplasmin; **HAP**, Haptoglobin; **HAPc**: Cleaved β chains of HAP; **HPX**: Hemopexin; **LRG**: Leucine rich glycoprotein; **EOCa**: Epithelial ovarian carcinoma; **GOCa**: Germ-line ovarian carcinoma.

## Competing interests

The authors declare that they have no competing interests.

## Authors' contributions

YC performed the experimental work and analysis of the data presented. BKL shared his clinical expertise on ovarian cancer and supplied the serum samples for the study. SCP provided her expertise for the immunohistochemical analysis of biopsy samples and interpretation of the results. PSAR assisted in interpretation of the protein mass spectrometry data and contributed in the preparation of the manuscript. OHH conceptualized the study, its design and authored the manuscript.
